# A Case of Right Ventricular Failure Secondary to Acute Chest Syndrome Managed With Early Red Cell Exchange Transfusion

**DOI:** 10.7759/cureus.37729

**Published:** 2023-04-17

**Authors:** Abeselom Geletu, Josephine Emole, Alaa Abu Sayf, Oscar A Hinojosa, Alexandra Gastesi

**Affiliations:** 1 Internal Medicine, Henry Ford Health System, Detroit, USA; 2 Hematology and Medical Oncology, Henry Ford Health System, Detroit, USA; 3 Pulmonary Disease & Critical Care, Henry Ford Health System, Detroit, USA; 4 Internal Medicine, Henry Ford Health Systems, Detroit, USA; 5 Pulmonary Disease and Critical Care, Henry Ford Health System, Detroit, USA

**Keywords:** red blood cell exchange, sickle cell beta-thalassemia, right ventricular failure, acute chest syndrome, sickle cell disease complications

## Abstract

Patients with sickle cell disease are at risk of vaso-occlusive crises including acute chest syndrome (ACS) and pulmonary hypertension. ACS is a life-threatening complication of sickle cell disease and is associated with increased morbidity and mortality. It is known that pulmonary pressures increase during episodes of acute chest syndrome and may lead to acute right ventricular failure leading to increased morbidity and mortality. Given the paucity of randomized controlled trials, the management of ACS and pulmonary hypertension in the setting of a sickle cell crisis largely relies on expert opinion. We present a case of acute chest syndrome complicated by acute right ventricular failure that was managed with prompt red cell exchange transfusion with favorable clinical outcomes.

## Introduction

Acute chest syndrome (ACS) in patients with sickle cell disease is diagnosed based on a constellation of symptoms, as well as laboratory and radiographic findings. These patients typically present sickle-cell pain crises and develop ACS - a few days after hospitalization. ACS would be suspected when they develop fever, chest pain, hypoxia, cough, and wheezing on lung examination. In addition, laboratory values may reveal leukocytosis, and these patients may develop new lung infiltrates on imaging [1]. ACS, when associated with pulmonary hypertension, could result in acute right ventricular (RV) failure, contributing to increased morbidity and mortality [2,3]. Given the paucity of randomized control trials, there is no standardized treatment algorithm for treating ACS and its uncommon but potentially fatal complication of acute right ventricular failure [1,4]. Acute RV failure in this setting is commonly managed with the optimization of right-heart hemodynamics with preload reduction, pulmonary vasodilators, and inotropic agents. Although red cell exchange transfusion has been used to treat ACS, it is unclear if it plays a role in managing acute RV failure [5]. This report presents a case of ACS complicated by acute right ventricular failure managed with prompt red cell exchange transfusion resulting in a favorable clinical outcome.

## Case presentation

**Figure 1:**
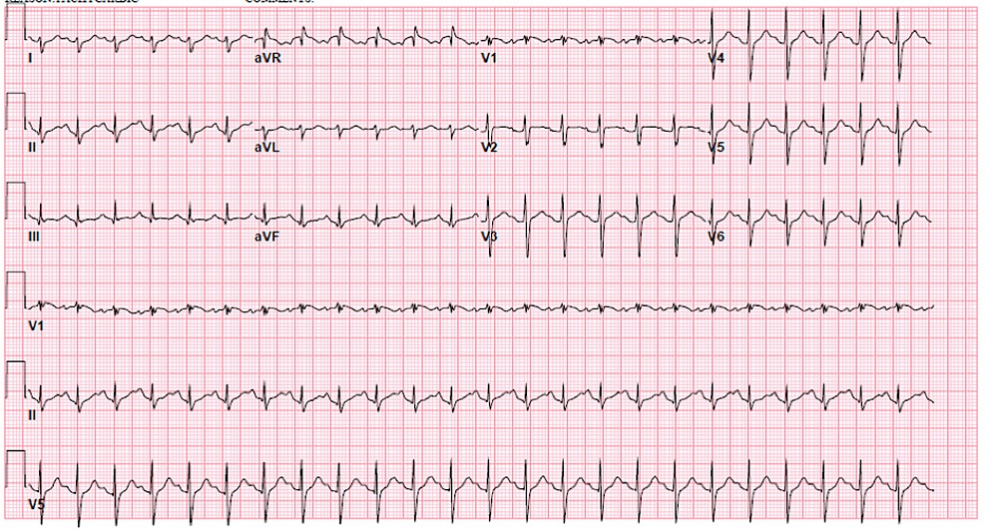
Electrocardiogram demonstrating right axis deviation

**Figure 2:**
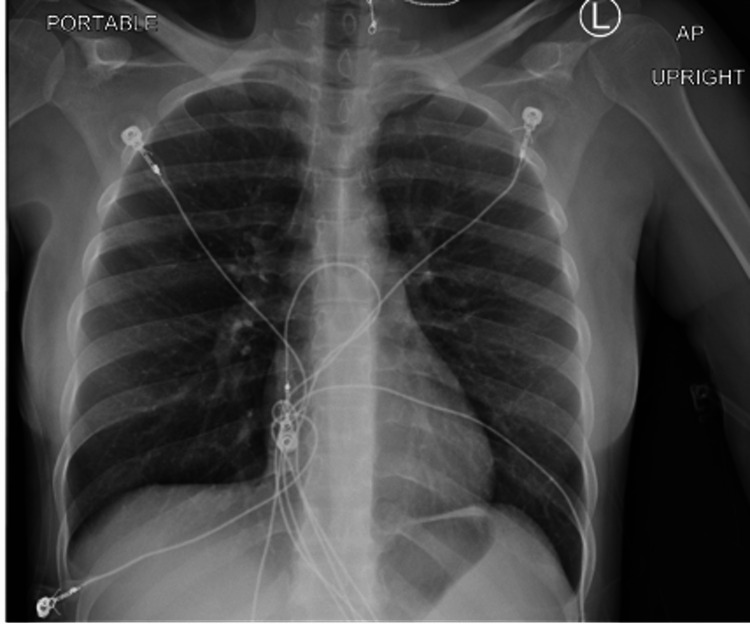
Chest x-ray on presentation without lung infiltrates

A 28-year-old female with a history of sickle cell presented with diffuse body pain, nausea, and decreased oral intake. Vital signs were notable for tachycardia (122 beats/minute), tachypnea (24 breaths/minute), and hypertension (143/95 mmHg). Physical examination was remarkable for tenderness of all four extremities. Electrocardiogram revealed sinus tachycardia with rightward axis deviation (Figure 1). The basic metabolic panel was notable for elevated beta-hydroxybutyrate. The liver profile revealed mild elevation of total and direct bilirubin. Venous blood gas analysis showed metabolic acidosis (Table 1). The initial chest x-ray did not demonstrate a new lung infiltrate (Figure 2). The patient was admitted to the observation unit for a sickle cell pain crisis and managed with intravenous hydration and a patient-controlled analgesia pump. On day two of presentation, she suddenly became febrile (maximum temperature of 39.3^o^C, tachycardic (148 beats per minute), tachypneic (22 breaths/minute), and hypoxic (83% on ambient air). Due to an acute change in mental status and an abrupt drop in hemoglobin (Table 2), she was transferred to the medical intensive care unit (MICU) for escalation of care. 

**Table 1:** Pertinent laboratory values mmol/L - millimoles Per liter; ng/L - nanograms Per Liter; mmHg - millimeters of mercury; mg/dL - milligrams per deciliter

	Beta-hydroxybutyrate	High Sensitivity Troponin	Lactate	Venous pH	Venous partial pressure of carbon dioxide	Anion gap	Bicarbonate	Total, Bilirubin	Direct bilirubin
Ref range & units	0.00 – 0.30 mmol/L	< 19 ng/L	<2.1 mmol/L	7.35 – 7.45	35 – 45 mmHg	3-13	22 – 26 mmol/L	<1.2 mg/dL	0 – 0.3 mg/dL
Day 1	4.31	6 and 6	1.0	7.34	33.1	16	17.2	2.6	0.6
Day 2	1.42	none	1.2	none	none	8	22	2.3	0.5
Day 3	none	none	1.9	none	none	10	30	2.2	0.4

**Table 2:** Pertinent hematological laboratory values Hb - hemoglobin; HbS, hemoglobin S; HbA1 - hemoglobin A1; HbA2 - hemoglobin A2; Hb C - hemoglobin C; Hb F - hemoglobin F; PLT - platelet count; WBC - white blood cell count; LDH - lactate dehydrogenase; g/dL - gram per deciliter; K/uL - thousands per cubic milliliter; IU/L - International units per liter; mg/dL - milligrams per deciliter

	HbS	HbA1	HbA2	Hb C	Hb F	Hb	PLT	WBC	LDH	Haptoglobin	Reticulocyte percent
Ref range & units	0.0%	96.5 – 97.8 %	2.2 – 3.2 %	0.0 %	< 2.0%	12.0 – 15.0 g/dL	150 – 450 K/uL	3.8 – 10.6 K/uL	<250 IU/L	30 – 200 mg/dL	0.5 - 1.5 %
Day 1	51.6	35.4	3.7	0.0	9.3	10.4	81	15.3		<30.0	4.4
Day 2	5.4	91.9	2.7	0.0	0.0	7.6	74	13.1	1190	<30.0	none
Day 3	none	none	none	none	none	8.8	36	13.1	3331	<30.0	5.3

**Figure 3:**
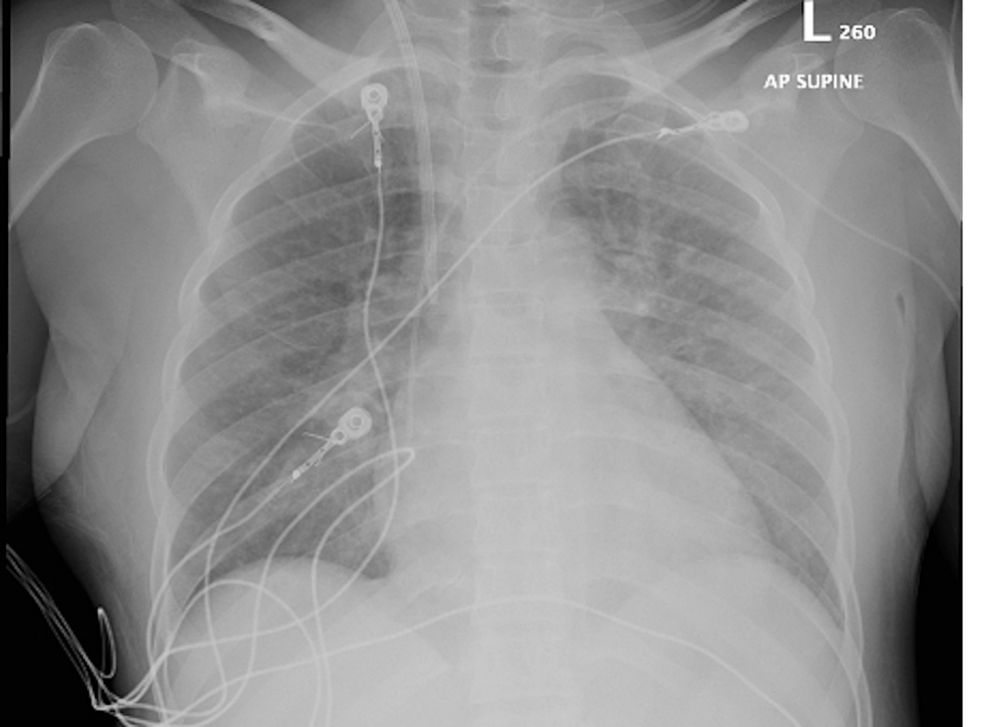
Chest x ray on day 2 of presentation showing patchy opacities int the left lung.

While in the MICU, a chest x-ray revealed new airspace opacities (Figure 3).

**Figure 4:**
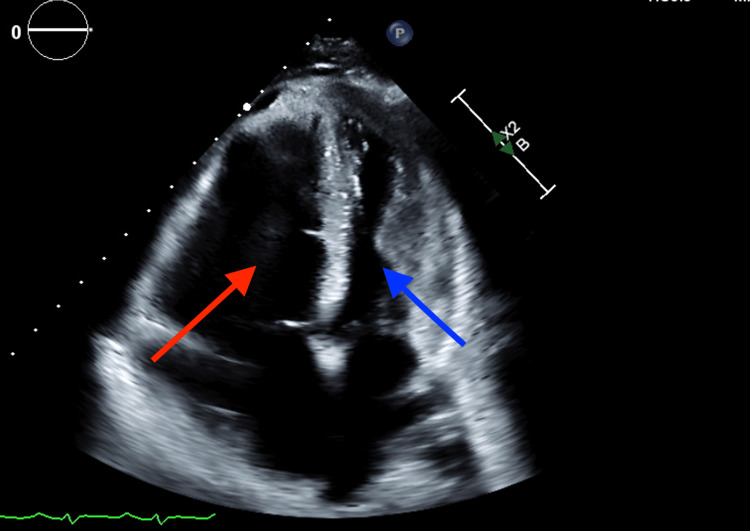
Transthoracic echocardiography showing McConnell's sign with severely dilated right ventricle (red arrow) with free wall hypokinesis and apical sparing and left ventricle (blue arrow).

**Figure 5:**
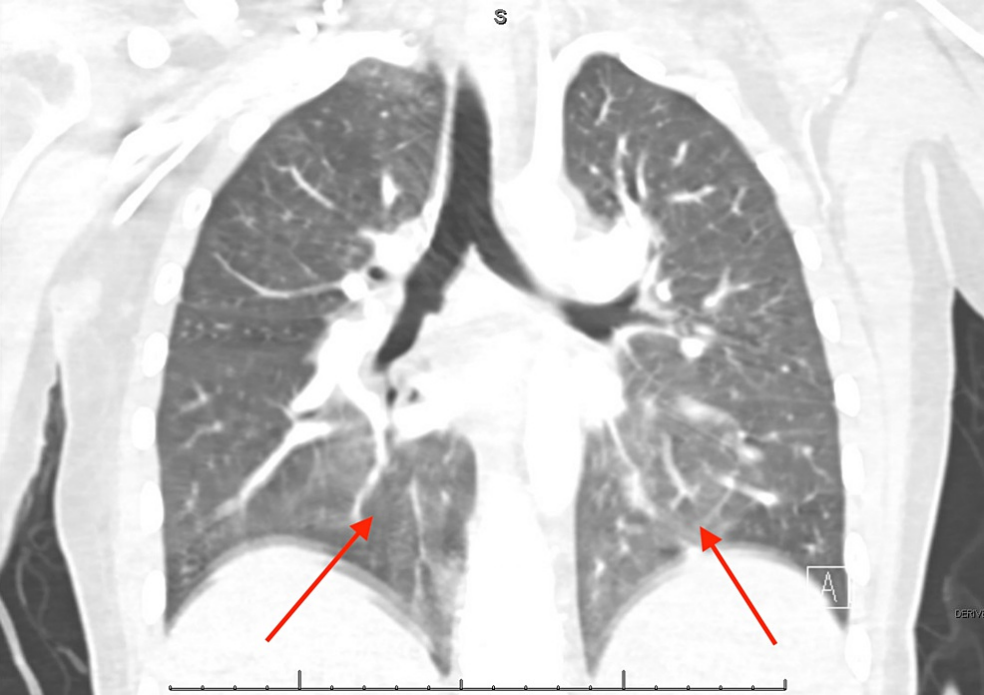
Computed tomography (CT) chest showing patchy ground glass opacities throughout the lower lobes Red arrows show new ground glass opacities

Given acute decompensation with sudden hypoxia, worsening tachycardia, and physical examination notable for jugular vein distention, an echocardiogram was obtained, which revealed a ‘D’ shaped left ventricle in diastole, McConnell’s sign, and severely reduced global right ventricular systolic function (Figure 4). A computed tomography scan was negative for pulmonary embolism but revealed ground glass opacities in the lower lobes (Figure 5). The overall presentation was consistent with acute chest syndrome. Empiric antibiotics were initiated with vancomycin and ceftriaxone, and a red cell exchange transfusion was planned, given the rapid clinical deterioration. She underwent one round of therapeutic exchange red blood cell transfusion with eight units of packed red blood cells. Following this, her oxygen requirement dramatically improved, and she was weaned down from 9 liters/minute nonheated high flow to a 2 liter/minute nasal cannula. In addition, the patient’s follow-up hemoglobin evaluation showed improved Hb S levels (Table 2). Follow up echocardiogram three days later demonstrated normal right ventricular size and mildly reduced global right ventricular systolic function. However, her course was further complicated by ischemic stroke leading to left lower extremity weakness, which mildly improved after the exchange transfusion and patient was discharged to inpatient rehabilitation facility.

## Discussion

The pathophysiology of acute chest syndrome (ACS) and consequent acute cardiopulmonary dysfunction is not fully understood. Still, it involves a complex interaction among several factors, including infection, inflammation, and vaso-occlusive crisis [2]. Severe vaso-occlusion could lead to pulmonary infarction, acute elevation of pulmonary pressures, and increased right ventricular afterload, resulting in acute right ventricular failure [5,6] and hemodynamic instability. Pulmonary hypertension can also result from a chronic state of hemolysis, as seen in sickle cell disease, resulting in pulmonary vasoconstriction [7,8] and exacerbated in vaso-occlusive crises [1]. In this report, we present a case of acute right ventricular failure in a patient with ACS and right heart ventricular dysfunction, whose clinical status significantly improved after red cell exchange transfusion, averting potential respiratory failure and cardiogenic shock. 

Laboratory, imaging findings, and treatment course, in this case, mirror what has been reported in the literature. As shown in Table 2, our patient had elevated lactate dehydrogenase (LDH), reticulocyte percent, and decreased haptoglobin, consistent with a state of hemolysis. There are reports that patients with acute chest syndrome can have evidence of right ventricular failure with echocardiogram findings of McConnell Sign [9] which was also demonstrated in our case. The cornerstone of treatment in ACS involves the use of supportive measures that address its multifactorial etiology: antibiotics, supplemental oxygenation, rehydration, and simple or red cell exchange transfusion [10]. Red cell exchange transfusion aims to minimize the amount of hemoglobin S, thereby increasing oxygenation and preventing further complications relating to poor oxygenation [11]

Given the paucity of randomized controlled studies, the decision to use blood transfusion in ACS is usually left to the discretion of the clinician [1]. Similarly, there are no clear guidelines about the use of red cell exchange transfusion in the treatment of ACS complicated by acute right ventricular failure. In general, management of right ventricular failure in these cases involves optimizing right-sided volume overload, use of pulmonary vasodilators, and inotropic agents in refractory cases [5].

Pulmonary hypertension is a frequent occurrence in sickle cell disease and acute vaso-occlusive crisis [4]. In this case, we opted not to conduct a right heart catheterization to better assess pulmonary pressures due to the patient's significant improvement following the exchange transfusion, which is the limitation of this case report. Nevertheless, it would have been advantageous to obtain data from a right heart catheterization to determine if there was an elevation in pulmonary hypertension during the acute chest syndrome, which led to an acute increase in right ventricular afterload and subsequent right ventricular failure. Furthermore, although explaining how red cell exchange transfusion improves right ventricular dysfunction in acute chest syndrome is not the main focus of this case report, it's possible that this is because red cell exchange transfusion reduces the quantity of Hemoglobin S, which in turn enhances oxygenation resulting in reduced pulmonary vasoconstriction [11]. 

Overall, our report demonstrates that the red cell exchange transfusion procedure should be considered early for ACS complicated by right ventricular failure.

## Conclusions

Our case highlights acute chest syndrome as a serious complication of sickle cell disease which demands prompt recognition and treatment. This syndrome is usually conservatively managed with pain control, rehydration, antibiotics, and supportive transfusions. There are, however, no clear guidelines for red cell exchange transfusion in acute chest syndrome complicated by acute right heart dysfunction. We describe our experience in the successful use of red cell exchange transfusion in acute chest syndrome to reverse right heart dysfunction and potentially fatal cardiopulmonary failure. Although this is a single case report, it would suggest that further studies should be conducted in exploring the utility of red cell exchange in patients who present with acute right heart dysfunction in the setting of acute chest syndrome. 
